# Patient Falls in Seclusion Rooms in Psychiatric Inpatient Care: A Sociotechnical Probabilistic Risk Modeling Study

**DOI:** 10.1097/NCQ.0000000000000683

**Published:** 2022-12-07

**Authors:** Jaakko Varpula, Maritta Välimäki, Johanna Pulkkinen, Tella Lantta

**Affiliations:** Department of Nursing Science, University of Turku, Turku, Finland (Mr Varpula and Drs Välimäki and Lantta); Xiangya Center for Evidence-Based Practice and Healthcare Innovation, Central South University, Hunan, China (Dr Välimäki); Hospital District of Southwest Finland, Turku, Finland (Dr Pulkkinen); and Department of Nursing, Faculty of Health and Education, Manchester Metropolitan University, Manchester, United Kingdom (Dr Lantta).

**Keywords:** accidental falls, hospital psychiatric department, inpatients, patient safety, risk assessment

## Abstract

Patient falls are a major adverse event in psychiatric inpatient care.

**Purpose:**

To model the risk for patient falls in seclusion rooms in psychiatric inpatient care.

**Methods:**

Sociotechnical probabilistic risk assessment (ST-PRA) was used to model the risk for falls. Data sources were the research team, literature review, and exploration groups of psychiatric nurses. Data were analyzed with fault tree analysis.

**Results:**

The risk for a patient fall in a seclusion room was 1.8%. Critical paths included diagnosis of a psychiatric disorder, the mechanism of falls, failure to assess and prevent falls, and psychological or physical reason. The most significant individual risk factor for falls was diagnosis of schizophrenia.

**Conclusions:**

Falls that occur in seclusion events are associated with physical and psychological risk factors. Therefore, risk assessment methods and fall prevention interventions considering patient behavioral disturbance and physiological risk factors in seclusion are warranted.

Seclusion in psychiatric inpatient care is used to improve the safety of patients and staff.^1^ Seclusion is a coercive intervention where a patient is isolated in a designated locked room.^2^ Studies have identified adverse events related to patient seclusion such as self-harm, suicide, and somatic complications.^3^ Yet, very few studies have explored whether patient falls are a considerable adverse event in patient seclusion.^4^ Generally, patient falls in psychiatric inpatient care are major adverse events that lead to injuries and even death.^5^ The fall rate of psychiatric patients ranges from 1.1 to 7.97 in adult psychiatric inpatient care to 17.7 in psychogeriatric care per 1000 patient days.^6^–^8^

The risk for falls in psychiatric inpatient care is typically assessed through a screening upon admission using various risk assessment tools. These assessments are normally conducted with tools developed for the general nonpsychiatric patient population.^9^ Risk assessment tools have also been developed specifically for the psychiatric population.^10^ However, the clinical usefulness of these tools with secluded patients is unclear. They are intended to be used during patient admission and may not be suitable for the quickly changing scenarios of seclusion and patients with severe behavioral disturbances.^11^

Currently, knowledge is scant on risk factors for falls, specifically for secluded patients. Known risk factors for patient falls in psychiatric care are patient diagnoses, such as depression,^12^ bipolar disorder,^13^ and psychosis.^14^ The use of psychotropic medication, sedatives, antidepressants,^15^ and cardiovascular medi-cation^16^ has been identified to increase the risk for falls. Other reported risk factors include physiological characteristics of patients, such as mobility, balance,^17^ and environment.^18^

Patient falls have typically been studied retrospectively to identify individual risk factors after the event has occurred, resulting in oversimplification.^19^ However, prospective methodologies such as sociotechnical probabilistic risk assessment (ST-PRA) can be used to minimize this shortcoming. ST-PRA considers contributions of human errors, behavior, technology, and process, all which are vital in studying risks in health care.^20^ This methodology was chosen as it has already been utilized successfully to predict falls in community living centers.^21^ Patient seclusion is complex, and more advanced methods are needed to assess the risk for falls. Therefore, the purpose of this study is to model the risk for patient falls in seclusion events in psychiatric inpatient care using ST-PRA methodology. The risk for falls is modeled by identifying risk factors and critical paths that lead to falls. The knowledge can be used to develop risk assessment and fall prevention interventions for secluded patients in psychiatric inpatient care.

## METHODS

### Methodological approach

This study employed ST-PRA methodology^22^,^23^ as the ST-PRA tool can be used to model multiple risk factors and their combinations for specific adverse events.^19^,^23^ Based on previous studies using ST-PRA, decisions can be made to prioritize and prevent specific risk factors.^23^ ST-PRA methodology includes 6 phases; see Table 1 for an overview of the phases.

**Table 1.** ST-PRA Methodology Phases Used in the Current Study

Phase	Aim of Phase	Overview of Phase	Data Sources
1	Identifying top event	Choosing the topic of interest, falls in seclusion (top event)	Research team, previous research
2	Identifying failure points	Identifying risk factors that contribute or are associated with falls in seclusion	Exploration group of psychiatric nurses, literature review, research team
3	Identifying dependencies, generating fault tree	Generating the fault tree and modeling the relationships and dependencies of risk factors for falls in seclusion	Research team
4	Validating fault tree	Validating the decisions made in the modeling process of the fault tree	Exploration group of psychiatric nurses, research team
5	Assigning probabilities	Assigning probabilities for each of the identified risk factors	Exploration group of psychiatric nurses, literature review, research team
6	Conducting sensitivity analysis	Conducting sensitivity analysis to ensure that conclusions from the model are appropriate	Research team

Abbreviation: ST-PRA, sociotechnical probabilistic risk assessment.

A key element of ST-PRA methodology is the fault tree, which is a graphical illustration of risk factors, their dependencies and relations, and how they contribute to the top event.^24^ The fault tree uses Boolean logic described with AND and OR gates. The AND gate indicates that 2 risk factors must occur together for a higher-level event to occur, whereas the OR gate means that a single risk factor by itself is sufficient to result in the higher-level event.^24^

### Setting

The study was conducted in a psychiatric inpatient care setting in Finland. According to the Finnish Mental Health Act (1116/1990), seclusion can be used, based on the decisions and assessment of the attending physician, to ensure the safety of patients, staff, and property.

### Data sources and collection

The data sources of this study were the research team, scientific literature, and exploration groups of psychiatric nurses. The research team consisted of researchers with a background in psychiatric nursing and experience with seclusion in psychiatric inpatient care and a researcher/clinician with experience in patient and occupational health and safety. The research team was involved in all phases of the study. They had an active role also as a data source as well as in the analysis.

A systematic literature search was conducted to identify risk factors and probabilities for patient falls using major health science databases (PubMed, PsycINFO, and CINAHL). Manual searches supplemented the systematic search. The literature review process is described in more detail in the Supplemental Digital Content Appendix (available at: http://links.lww.com/JNCQ/B62).

The exploration groups with psychiatric nurses were used to identify risk factors for falls (phase 2), validating the fault tree (phase 4), and assigning probabilities (phase 5). Participants for the exploration groups were selected using purposive sampling to reach individuals with experience with caring for patients in seclusion rooms in psychiatric inpatient care. Inclusion criteria for participants were voluntary participation, registered nurse, licensed practical nurse or charge nurse, permanent employment or work experience of at least 1 year, experience with using seclusion, and command and understanding of the Finnish language. Three exploration groups were organized between September 2021 and February 2022. The same participants were invited to all 3 exploration groups. However, not all participants could attend all 3 appointments due to their shift work. The exploration groups were audio recorded.

### Data analysis

Information about the characteristics of the included studies can be found in the Supplemental Digital Content Appendix (available at: http://links.lww.com/JNCQ/B62). Results of the studies were synthesized to provide an overview, including incidence of falls, risk factors for falls, and mechanism of fall as defined by each study. Data in phases 2, 4, and 5 were analyzed using direct coding, where risk factors identified by nurses and their probabilities were coded and added to the synthesis tables.

#### Generating the fault tree model

Data from the exploration groups, literature review, and research team discussions guided the generation and modification of the fault tree. Relyence (Relyence UK Limited) software was used to model the fault tree. A preliminary list of risk factors was recognized from the literature review and exploration groups (phase 2). Ad hoc searches were conducted for risk factors identified only in the exploration groups to justify their inclusion in the fault tree. Risk factors that require further investigation were defined as underdeveloped events. To generate the initial fault tree, the research team discussed how each risk factor could result in a patient fall. In the discussions, risk factors were classified into themes: patient behavior, the physical condition of the patient, influence of medication, and the seclusion environment. Once the risk factors were collated in themes, the research team discussed how these risk factors were related to and dependent on each other. The risk factors were modeled to the fault tree with AND and OR gates.

The fault tree was validated in phase 4 to confirm its logic and that it represents reality. The logic and connections of the fault tree were critically assessed in the exploration group. Modifications were made based on the feedback and discussion among the research team. Risk factors that were deemed unrelated or negligible were removed from the fault tree.

Probabilities for risk factors were assigned in phase 5. Probabilities from the literature review were assigned first since they provided the most reliable estimate. The literature did not have probability estimates for all risk factors. For those risk factors, we used probability estimates provided in the exploration group and research team discussions. Risk factors to which no probability estimate could be assigned were removed, as their contribution to falls could not be established. Relyence software was used to calculate probabilities for the top event and all gates. The software used Boolean algebra. For the AND gates, probabilities for risk factors were multiplied. For the OR gates, probabilities for risk factors were added together, and any overlap was subtracted so that it would not be counted more than once if both or all risk factors occurred at the same time.^24^ This calculation resulted in cut sets and total risk for fall in seclusion events.

The cut sets were the combination of risk factors resulting in a fall. In this study, we analyzed the minimal cut sets, which were the minimum combination of risk factors that formed a critical path that resulted in fall.^23^ The criticality of each individual risk factor was also assessed. In the results, we present the most critical minimal cut sets (see the Supplemental Digital Content Table, available at: http://links.lww.com/JNCQ/B63) and criticality of each individual risk factor (Table 2).

**Table 2.** Most Significant Individual Risk Factors Based on Average Estimates

Risk Factor	Criticality^a^
Getting out of bed	0.345884
From seated to standing position	0.334611
Schizophrenia	0.209765
Getting to bed	0.176398
Bipolar disorder	0.149798
Psychiatric comorbidity	0.132473
Alzheimer's	0.114458
Lewy body dementia	0.106042
Personality disorder	0.092496

^a^Criticality is the probability that the fall is a result of an individual risk factor.

We performed sensitivity analysis on risk factors where large variation was reported in the literature. We calculated the average of each of these risk factors to form the average estimate. For the low estimate, we calculated the average without the highest reported probability value. For the high estimate, the average was calculated without the smallest reported probability value. Further, we conducted a sensitivity analysis with and without the inclusion of elderly patients (older than 65 years) and history of medical problems as risk factors. These were removed after the sensitivity analysis because of their unspecified, yet significant, contribution to falls.

### Ethical considerations

The ethical review was conducted by the Ethics Committee for Human Sciences at the University of Turku (15/2021). The study organization approved this study. Data were made available after the completion of the study, according to the European Union recommendations for open science.^25^ The anonymized data were stored in a Zenodo open data repository^26^ (doi.10.5281/zenodo.7276312).

## RESULTS

The final sample included 31 articles from the literature review and 6 psychiatric nurses. The fault tree consisted of 88 risk factors for falls in seclusion. These risk factors were modeled in the fault tree with gates (22 OR gates, 2 AND gates). Of the risk factors, 12 were labeled as underdeveloped events.

The fault tree analysis resulted in an overall risk of 0.018026 (1.8%), meaning that 9 of every 500 patients fall in seclusion. In the development of the fault tree, we identified critical paths that may lead to a fall, including the specific mechanism of fall (eg, slipping), the disorder of the patient (eg, bipolar disorder), the failure to prevent a fall (where risk assessment and fall prevention intervention are conducted), and reason for the fall (psychological or physical). The critical paths are illustrated in the Figure.

**Figure.**
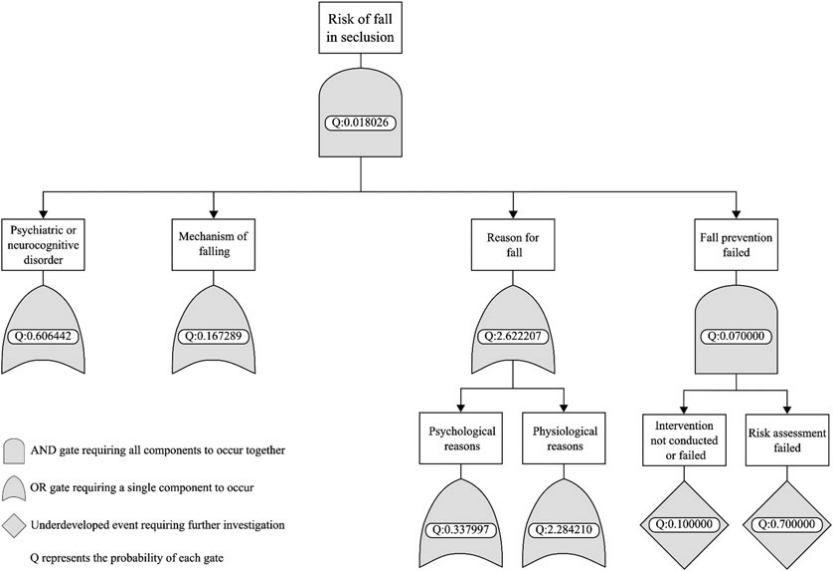
Critical paths.

Based on the minimal cut sets, a fall during seclusion requires a certain disorder, mechanism of fall, reason for falls, and a failure in fall prevention. Diagnoses of schizophrenia and bipolar disorder were most prevalent. The most notable mechanism of falls was when patients moved from the bed or seated position to a standing position. In the minimal cut sets with highest probabilities, the reasons were physiological: narrow base of support, obesity, or gait abnormality, and side effects of medication, such as dizziness, muscle weakness, and arrhythmia. The failure of fall prevention consisted of the failure of both risk assessment and fall prevention intervention.

The individual risk factors for falls are described in Table 2. The highest independent contributions to falls were when a patient mobilized out of the bed (0.345884) and when a patient went from a seated to a standing position (0.334611). The diagnosis that had the highest criticality was schizophrenia (0.209765) followed by bipolar disorder (0.149798) and Alzheimer's (0.114458).

## DISCUSSION

The aim of this study was to model the risk for falls for psychiatric patients in seclusion. The model resulted in an overall risk of 0.018 (1.8%) for falls in seclusion. The risk for falls is similar (0.079) to that of another study that used ST-PRA methodology to predict injurious fall risks for patients living in community centers.^21^ Despite the relatively low fall risk, the consequences of falls can be severe.^27^

The fault tree analysis enabled ranking the most important risk factors based on their contribution to falls. The most significant individual risk factors were specific diagnoses and physiological reasons. The impact of psychological risk factors, such as aggression and anxious behavior, were less significant. This is important because in seclusion the focus is more on the behavioral disturbance of the patient^28^ and less on their physical condition.^29^ The contribution of physical risk factors, such as physical impairments and side effects of medication to falls, is significant in both seclusion and in general psychiatric inpatient care.^30^ Therefore, more focus is needed on the physical risk assessment for secluded patients.

The high criticality of schizophrenia and bipolar disorders as individual risk factors could be because these diagnoses represent a large proportion of patients in psychiatric inpatient care as well as patients who are secluded.^31^ These patient groups also have many physiological conditions that increase their fall risk, especially cardiovascular diseases.^32^ According to our model and previous literature, the risk for falls is increased with medications, such as cardiovascular and psychotropic medications.^15^,^16^,^33^ Patients with psychological and physiological conditions need to be closely observed during seclusion due to their increased risk for falls.

Patients who have a high risk of falling due to physical and psychological risk factors are likely to be elderly. Although we did not include elderly patients in the model, many of the risk factors are prevalent in elderly patients,^34^ including gait abnormality,^6^ narrow base of support,^35^ and use of stool softeners.^13^ Therefore, for elderly patients with a high risk for falls, constant one-to-one observation may be more suitable than seclusion for fall prevention.

Fall prevention in psychiatric inpatient care has focused on fall risk assessment, which is not sufficient to reduce falls; it needs to be combined with fall prevention interventions.^36^,^37^ A recent review concluded that there is a lack of intervention studies of fall prevention in psychiatric settings. The existing interventions were found to concentrate on single aspects of fall prevention, such as exercise or making the environment safer. Furthermore, the studies focused on patients with cognitive impairments, such as dementia, instead of psychiatric patients.^38^ Fall prevention measures were not identified by members of the exploration group of this study either, highlighting the need for developing fall prevention interventions for psychiatric patients. Physical and psychological factors, as well as the context of seclusion, need to be considered in the development of these interventions, as patients with severe symptoms are most often secluded.^39^ The seclusion environment holds risk factors for tripping, slipping, and stumbling for patients who have mobility difficulties or have been sedated. Small environmental changes such as floor materials, lighting, and removal of doorsteps could help reduce risk factors for falls as extrinsic, environmental factors account for a significant proportion of falls.^40^ The findings of this study can be used to provide a basis for such interventions; the model can be used to steer the focus on the most significant risk factors. In fall prevention and risk assessment, the role of nurses is important as they are responsible for the care of secluded patients. Thus, to increase nursing care quality by preventing falls, regular observation and assessment are warranted for secluded patients.

The model should be further developed to improve its reliability. Further developments require probability estimates derived from using a combination of different methods. Most of the studies included in the review relied on information from incident reports. Previous studies have shown that incident reports result in the underreporting of adverse events^41^ including falls.^42^ Approximately 10% of incident reports provide information regarding the etiology of adverse events.^43^ To compensate for these shortcomings, medical record reviews and text mining approaches could be used to identify risk factors^44^ to strengthen the model's reliability.

### Limitations

The study includes limitations. First, we aimed to include patients as their contribution would have been valuable in recognizing risk factors that the literature and professionals can overlook.^45^ However, we were unable to recruit any patients. This might be because seclusion events can be traumatic,^3^ and participating may have caused negative experiences to resurface. Second, probabilities for risk factors were derived from the literature and professional experience. For some risk factors, the probability estimates relied only on individual studies. The probability estimates from professionals may not be precise, but they are more valuable than no estimates at all.^23^ Due to the limitations of the literature, the impact of time exposure on probabilities could not be assessed to determine whether increased time in seclusion increases the risk for falls. Third, the fall risk and risk factors were assigned prospectively; therefore, retrospective studies on falls in seclusion are required to assess whether the conclusions made in this study are accurate. Fourth, some significant risk factors were defined as underdeveloped events; these risk factors are significant and require further research. Last, the literature sources focused on falls in psychiatric inpatient care; previous studies on falls in seclusion do not exist. Secluded patients have more severe behavioral disturbances^11^; as such, probabilities assigned from the literature on general psychiatry might underestimate the risk for patient falls in seclusion.

## CONCLUSIONS

A significant proportion of risk factors are associated with the physical condition of the patients, behavioral disturbances, and the seclusion environment. Falls can lead to severe injuries and can have detrimental consequences, even death. Therefore, a fall risk assessment method that considers patients with behavioral disturbances in seclusion is needed. Furthermore, fall prevention strategies and interventions for psychiatric inpatient population are needed.

## Supplementary Material

SUPPLEMENTARY MATERIAL
