# Supplementary material for: Patient Falls in Seclusion Rooms in Psychiatric Inpatient Care: A Sociotechnical Probabilistic Risk Modeling Study
Source: J Nurs Care Qual. 2022 Dec 7;38(2):190–7. doi: 10.1097/NCQ.0000000000000683 (PMC9944373; doi:10.1097/NCQ.0000000000000683)
Supplement: SUPPLEMENTARY MATERIAL [file jncqu-38-190-s001.docx]

**Supplementary Digital Content Appendix.** Literature review

A systematic review was conducted following the Preferred Items for Systematic Review and Meta-Analysis Protocols (PRISMA-P) guideline (Shamseer et al. 2015).

**Study selection criteria**

*Types of studies:* Empirical studies of quantitative, qualitative, or mixed methods design were included (Pluye et al. 2016). Abstracts, posters, thesis, and dissertations were also included. Publications were excluded if they were editorial, description of an intervention without data, methodological paper or history of a case (Pluye et al. 2016).

*Types of participants*: Included participants were adults (over 18 years of age) treated in psychiatric inpatient care (Adult general, psychogeriatric, forensic psychiatric). Patients with all types of psychiatric diagnoses were included. Studies including patients with intellectual disabilities were included if they are treated in psychiatric inpatient care. Studies with patients under the age of 18 years were included only if they were treated in adult psychiatric inpatient care. Studies were excluded if they focused on adolescent psychiatric inpatient care, specialized units for intellectual disabilities, or elderly care.

*Types of outcome measures:* Incidence of fall, risk of fall, mechanism of fall as defined by the study during psychiatric inpatient care. Prevalence, descriptions of risks and mechanism of falls were included.

**Information sources and search strategy**

A literature search was conducted in three bibliographic databases: PubMed, PsycINFO (EBSCO), and CINAHL (EBSCO). The electronic search was supplemented with a manual search from the reference lists of selected studies and a general search using Google Scholar. The search terms used included wide range of subject headings, keywords, and phrases. PICO framework was used in formatting the search terms (Miller & Forrest 2001). Each of the search terms had multiple variations. The relevant formation for each of the selected databases was used. The search terms were developed together with research librarian. The full search string for PubMed is presented in table 1.

Table 1 PubMed search string

| PubMed  ("psychiatric hospital*" OR "psychiatric care*" OR "psychiatric unit*" OR "psychiatric nursing*" OR "psychiatric ward*" OR "psychiatric service*" OR "psychiatric setting*" OR "mental health ward" OR "mental health wards" OR "mental health settings" OR "mental health setting" OR "mental health unit" OR "mental health units" OR "psychiatric institutional care" OR "forensic psychiatr*" OR "forensic setting*" OR "Hospitals, Psychiatric"[Mesh] OR "Forensic Psychiatry"[Mesh] OR "Mental Health Services"[Mesh] OR "Mental Health Service" OR "mental health services" OR "Psychiatric Nursing"[Mesh])  AND  (patient OR patients OR "Patients"[Mesh] OR "vulnerable adult*" OR "Vulnerable Populations"[Mesh] OR "vulnerable population*" OR inpatient* OR "Inpatients"[Mesh] OR "psychiatric patient*" OR "mental health patient" OR "mental health patients" OR consumer* OR client* OR "service user*")  AND  (fall* OR "fall risk" OR "fall mechanism" OR "inpatient fall" OR fall-risk OR "Accidental Falls"[Mesh] OR fell OR "patient fall*")  AND  ("risk of injury" OR "injury risk*" OR "risk for injury" OR wound* OR "Wounds and Injuries"[Mesh] OR "wounds and injury" OR "Wounds and Injuries" OR "adverse effects" [Subheading] OR "adverse effect*" OR "Long Term Adverse Effects"[Mesh] OR "adverse impact*" OR "adverse event*" OR "adverse incident*" OR harmful* OR harm OR harms* OR "patient safety incident" OR "patient safety" OR "safety hazard*" OR hazard* OR incident*) | 1344 |
| --- | --- |

**Screening and data extraction**

One reviewer JV performed the selection of studies independently. A reference management software Mendely was used. First, duplicate studies were removed. Second, studies were excluded based on title/abstract review. Third, full-texts of studies were assessed for eligibility. One researcher (JV) extracted the information from the selected studies (study characteristics and outcome data).

**Data items**

Information of interest from the studies included study characteristics: purpose of the study, country, design, methods, sample, age, and diagnosis. Study results on the incidence of fall, risks for fall, and mechanism of fall.

**Data synthesis**

The search results are summarized in the PRISMA flow chart (Moher et al. 2010), figure 1. Information from the data sources (study characteristics) were recorded in a spreadsheet (table 1.), the information recorded was: purpose, country, design, methods, sample, age, and diagnosis.

Second, iterative intra-method synthesis was conducted, qualitative and quantitative studies were separated, and mixed methods studies were fractioned (their qualitative and quantitative evidence are divided into their distinct components), and synthesized separately from each other. This provided a separate overviews and findings of both qualitative and quantitative datasets. Quantitative studies were synthesized and presented in a table 2. information synthesized were: incidence of falls, risks, and mechanism of fall as defined in the study. Qualitative findings were synthesized and summarized in table 3.

**RESULTS**

Total of 1906 potentially eligible studies were identified. Of these 432 were excluded as duplicates. 1474 studies were screened by title and abstract, out of these 1349 were excluded. Full text screening was conducted for 125 studies, of which 94 were excluded. 31 studies were included in the synthesis. Furthermore, ad hoc searches were conducted for specific risk factors to justify their inclusion and to assign a probability estimate. Ad hoc searches identified 9 studies.

Full-text articles excluded,
(n = 94)

## Included

## Eligibility

## Screening

## Identification

Records after duplicates removed
(n = 1474)

Studies included synthesis
(n = 31)

Full-text articles assessed for eligibility
(n = 125)

Records screened for title and abstract
(n = 1474)

Additional records identified through other sources
(n = 9)

Records identified through database searching
(n = 1897)

Records excluded
(n = 1349)

Figure 1 PRISMA flow chart (Moher et al. 2010).

**Table 1.** Study characteristics

| Study | Purpose | Country | Design | Methods | Sample | Age range, years (median) | Diagnosis |
| --- | --- | --- | --- | --- | --- | --- | --- |
| Vaughn et al. 1993 | To identify variables associated with psychiatric patient falls and to use that information to assess risk and, therefore prevent falls in this population | USA | A retrospective descriptive study | A data collection instrument was used in incident reports. Descriptive statistics. | Incident reports of falls (n=52) (38% male, 62% female) | 16 to 86, Mean 55,3, mode 67. | Depression (n = 11, 30%), Bipolar Disorder (n = 9, 24%), Schizophrenia (n=3, 8%), Organic disorder (n=2, 5%). |
| Hawton & Leopoldt 1978 | To determine the frequency, nature and severity of accidents among psychiatric hospital in-patients, identify patients particularly in risk. | UK | Survey | Survey of routine accident form | All patients in acute, long-stay, and rehabilitation wards. 372 accidents of 161 patietns. | Not reported | Not reported |
| Yates & Tart. 2012 | To comparatively analyze falls in psychiatric and medical inpatient populations prior to, during and after revisions were made to the Policy. Nursing knowledge and perceived effectiveness of the revised fall prevention interventions were assessed. | USA | Retrospective and comparative design. | Collecting patient fall data with Fall Evaluation Tool (FET) from organization’s medical record database. Statistical analysis. | Psychiatric and medical patients over 18 years of age who fell during the data collection. Phase 1 (n =87) (psychiatry n=62) Phase 2 (n = 95), psychiatry (n=42). | Phase 1 (psychiatry) 21-90 years (M 59.34). non-geriatric (n=41), geriatric (n=21)  Phase 2 (psychiatry) 23-88 (M 58.21). Non-geriatric (n=25), geriatric (n=17). | Not reported |
| Aso & Okamura 2018 | To determine the risk factors for falls with a focus on balance among patients with schizophrenia hospitalized in the psychiatric ward | Japan | Prospective cohort design | Assessment of patients with schizophrenia in baseline with postural sway and maximum step length (MSL), follow-up of fall events. Statistical analysis | Patients with schizophrenia (N=120) during a 3-month follow-up. 60% female. | Mean 64.6 (SD 14.8), range 34-89. | Schizophrenia |
| Scanlan et al. 2012 | To explore factors associated with falls reported in inpatient psychiatric units in a metropolitan mental health service | Australia | Not reported | Incident reports were independently reviewed to determine contributory factors using an established coding methodology. | Incident reports (N=559) of psychiatric units (N=24) with 445972 occupied bed days. | Not reported | Not reported |
| Suga et al. 2019 | To examine differences in the patterns and risk factors for falls between younger and older patients at a psychiatric hospital in Japan | Japan | Retrospective observational study | Incident reports with demographic variables, time, severity and description of falls. Falls were classified by severity of injuries. Statistical analysis and coding of descriptive data. | Patients (N=379) of which younger (N=123), older (N=256). Male (n=200, 52.8%), female (n=163, 43%). | All fallers 71 (SD 14.3), range 31-98. | Organic mental disorder (n=223, 58.8%), schizophrenia (n=99, 26.1%), mood disorder (n=24, 6.3%), intellectual disability (n=22, 5.8%), others (n=10, 2.6%). |
| Struble-Fitzsimmons 2018 | To determine if the Timed Up and Go Test is a predictive tool to identify high fall risk patients in the inpatient geriatric psychiatry setting | USA | Retrospective chart review using a between groups design | Medical record review of patient age, gender, fall history, Timed Up and Go Test score, assistive device use. Statistical analysis. | Total sample (N=62) female (n=45, 72.6%),male (n=17, 27.4%). Non-fallers (n=33), fallers (n=29). | Fallers mean age was 75.79 (SD 9.60), range 60-97. | Not reported |
| Tsuji et al. 2017 | To analyze the factors causing falling with schizophrenia | Japan | Not reported | Patients muscle strength, balance, walking, flexibility, sleeping, antipsychotic drug intake and number of falls were assessed | Sample (N=19) consisted of fall group (n=12), male (n=4), female (n=8). | Age mean 64.5 (SD 5.4), range 53-72. | Not reported |
| Meng et al. 2010 | To identify psychiatric patients who are at high risk of falls. | Singapore | Retrospective design | Incident reports of falls and medical record review. | Patients (n=26), male (n=12), female (n=14). | Below 20 (n=3), 20-29 (n=8), 30-39 (n=1), 40-49 (n=1), 50-59 (n=2), 60-69 (n=5), 70-79 (n=3), 80 and above (n=3). | Eating disorders (n=8), Mood disorders (n=17), Psychotic disorders (n=2), Sleeping disorders (n=1), Dementia (n=4), Addiction (n=2). |
| Blair & Gruman 2006 | To quantify the number and types of falls and identify associated risk factors | USA | Descriptive design | Data collected with hospital-based fall prevention protocol assessment tool. | Total of patients (N=174). Most were female (n=100, 57.5%). | Average age was 74.1 (SD 9.78). | Top five diagnoses were major depression (n=81, 81%), dementia (n=43, 43%), schizoaffective disorder (n=20, 20%), bipolar disorder (n=19, 9.5%), schizophrenia (n=12, 12%). |
| Healey et al. 2007 | To describe the documented characteristics of reported accident falls with respect to frequency, related harm, timing, age and gender | UK | Retrospective descriptive design | Reports of a 6-month period, analysed with statistical methods | Total of fall reports (N=206350) | Age were reported in (n=108360) falls. 82.6% were over the age of 65, and 67.5% of patients over age of 75, and 32.4% over 85. | Not reported |
| Oepen et al. 2018 | To determine the fall rate and to analyze fall-related injuries | Germany | Retrospective analysis of fall protocols | Collection of incident reports and statistical analysis | Total patients (N=853) of which single fallers (n=125), and multiple fallers (n=92). | of Single fallers mean age was 80 (SD 8), and multiple fallers 79 (SD 8). | Single fallers (SF), and multiple fallers (MF) dementia in Alzheimer’s (SF n=49, 40%) (MF n=41 45%), Dementia in non-Alzheimer’s (SF n=23, 18%), (MF n=25, 27%) delirium (SF n=16, 13%), (MF n=10, 11%) organic affective disorder (SF n=2, 2%), (MF n=0, 0%) Schizophrenia (SF n=8, 6%), (MF n=6, 6%), mood disorder (SF n=23, 18%), (MF n=7, 8%). |
| Tsai et al. 1998 | To document risk factors for falls and their circumstances surrounding falls. | USA | Retrospective comparative design | Data collection over a 7-month period with two instruments (Risk Factors Checklist, Fall Description Form). | Patients (N=197) of which patients fell (n=17) (women 68%). | The average age was 50.9 years, range 18-91. | Major depressive (n=4, 21%), borderline personality disorder (n=4, 21%), schizoaffective disorder (n=3, 16%), psychotic depression (n=2, 11%), bipolar affective disorder (n=2, 11%). |
| Nyberg et al. 1997 | To prospectively investigate the incidence and consequences of falls in three types of geriatric care institutions | Sweden | Prospective study | Observation of 77 days with reporting of all fall incidents. Statistical analysis methods. | Total falls (N=299) of which (n=162) occurred in psychogeriatric clinics with 59% women. | The average age of patients in psychogeriatric clinic was 77.7 (SD 7.6). | Not reported |
| Furness et al. 2017 | To describe fall episodes by developing a post-fall reporting too of intrinsic and extrinsic fall risk factors specific to older persons with SMI. Describe fall risk factors and predictros of risk based on people who fell during 18-month period | Australia | Observational prospective cohort study | A post-fall reporting tool was developed. intrinsic and extrinsic fall risk factors over  18-month period. Descriptive and inferential analyses. | Total of patients (N=482) of which fallers (n=70, 14.5%). of fallers male (n=26, 37.1%) | Average age of fallers 78.2 (SD 6.9). | Dementia 54%, depression 25.7%, psychotic illness 30%, other diagnosis 4.3%. |
| Ocker et al. 2020 | The quality improvement project aims to reduce the rate of fall-related injuries on the adult inpatient behavioral health unit. To describe the  redesign of a fall prevention program | USA | Quality improvement design | Root cause analyses (RCAs) three fall-related major injuries. Literature review to identify EBP recommendations for fall prevention on behavioral health units. A fall  prevention action program was developed | Not reported | Not reported | Not reported |
| Jones et al. 1991 | To examine the relationship between halo patient characteristic and patient falls | USA | Retrospective comparative design | Collecting of incident reports and statistical analysis between fallers and control groups | The sample of psychiatric patients’ fallers (n=96), and non-fallers (n=100). | Patient age group for fallers 1-12 (n=10, 10.4%), 13-19 (n=25, 26%), 20-39 (n=10, 10.4%), 40-59 (n=22, 23%), 60+ (n=29, 30.2%). | Of the fall group schizophrenia (n=9, 9.5%), affective disorder (n=36, 37.9%), neurotic personality (n=11, 11.6%), substance dependence (n=26, 27.4%), childhood disturbances (n=9, 9.5%), other (n=4, 4.2%). |
| Abraham. 2012 | To explore psychiatric unit directors’ perceptions of the factors that contribute to patient falls in the psychiatric units | USA | Cross-sectional descriptive design | Online survey with statistical analysis | Surveys (n=66) of psychiatric nursing directors. | Age of participants was 25-34 (n=2, 2%), 35-44 (n=9, 13.6%), 45-54 (n=24, 36.4%), 55-64 (n=30, 45.5%), over 65 (n=1, 1.5%) | Not reported |
| Irvin. 1999 | To investigate if the variables from literature contribute to fall events on this unit | USA | Not reported | Fall Risk Assessment instrument used to patients admitted on a psychiatric unit. A chart review was conducted. | Patients (N=337) admitted to the psychiatric unit. | The mean age of the fall group was 67.8 (SD 18.5) range 20-93. | Not reported |
| Draper et al. 2004 | To identify patients at high risk of falling in Aged Care Psychiatry Unit by developing a falls risk assessment tool. | Australia | Not reported | Consecutive admissions were assessed. with ACPU Falls Screen, HoNOS 65+, RUG ADL, other information from medical records. Statistical methods | Total admissions (N=100), of patients (n=81). | Mean age was 78.6 (SD 7.4) | Depression (n=40, 42%), dementia (n=27, 28%), schizophrenia (n=12, 13%), delirium (n=5, 5%), organic disorders (n=2, 2%), drug and alcohol (n=1, 1%), other (n=8, 8%). |
| Chan et al. 2013 | To  timely identify all potential risk factors associated with falls and fall-related injury in a psychiatric  inpatient setting | Taiwan | Retrospective case control study | Patients with fall and controls were recruited. Medical record review was conducted. Risk assessment was conducted after falls. Statistical analysis. | Patients with falls (n=145), and controls (n=145). Patients with falls were male (n=62, 42.8%). | Patients with falls age was 47.4 (SD 13.3). | Patients with falls had schizophrenia (n=112, 77.2%), bipolar disorder (n=20, 13.8%), psychotic disorder due to general medical condition (n=13, 9%). |
| Bayramzadeh et al. 2019 | To describe the spatial and temporal pattern of falls occurrences and their location in  relation to the levels of safety continuum model | USA | Exploratory case study design | Retrospective data on patient falls were collected and analyzed. Data focused on extrinsic factors for falls, emphasizing the physical  environment. Content analysis. | Total of inpatient falls (N=818) of 49.7% (n=325) female. | Not reported | Not reported |
| Lee et al. 2012 | To identify how falls on psychiatric units occur, the underlying root causes and effective action plans to reduce  falls and injuries. | USA | Root Cause Analysis | A search of the Veterans Health Administration National Center for Patient Safety database was conducted to identify root cause analysis  (RCA) reviews where a fall was sustained by a patient on a psychiatric unit. | Total of 75 RCA were included. | Not reported | Not reported |
| Knight & Coakley. 2010 | The overall purpose of this quality improvement  initiative was to identify factors associated  with fall risk in a nonelderly psychiatric  population aged 18 to 65 years | USA | Retrospective | Review of medical charts for patients who fell. | Falls (N=14) male (n=2), female (n=12). | Mean 42 (range 21-65) | Bipolar manic (n=5); bipolar  mixed (n=1); bipolar depressed (n=1); schizoaffective  disorder (n=4); schizophreniform disorder  (n=1); and psychosis not otherwise specified  (n=2). |
| Dickinson et al. 2014. | To explore how fall risk, prevention and management  is understood and experienced in everyday practice by patients, family  carers and staff, in inpatient mental health settings providing care for  older people and (b) to identify how assessment tools, guidelines and  policy for fall prevention are used in these care environments | UK | A case study approach | Documentary analysis, Non-participant observation, analysis of fall incident reports, patient tracking was used to explore patient’s  experiences of a fall | Falls (N=920). 57% female. | Average age 81.7 years (range 59 to 99 years; SD 8.3) | diagnosis 40% of fallers had a primary functional diagnosis, 46% an organic mental health  diagnosis (14% non-specific diagnosis). |
| Brown & Edelstein. 2000 | One purpose of the  present study was to identify significant intrinsic predictors of falls  among hospitalized psychogeriatric residents and determine the accuracy  with which a combination of these variables can classify fallers  and non-fallers. The second purpose of the study was to determine  whether particular aspects of one’s environment set the occasion for  falling. | USA | A retrospective comparative study | Fall information was  collected from fall/incident forms and medical records.  Logistic regression to predict fallers and non-fallers. | Participants (N=144). Females (n=79, 55%), males (n=65, 45%). | Mean age 73.33, (SD 8.57). | Psychotic disorder (40%) dementia (27%), organic mental disorder (8%), mental retardation (6%), bipolar disorder (6%), depression (1%), other psychiatric disorder (15%), non-psychiatric disorder (19%). |
| Feng-Rong et al. 2009 | To investigate (a) the rate  of falls in a large psychiatric hospital in Beijing, China,  and (b) the risk factors for falls | China | Retrospective comparative study | Chart  review, The comparison between  the patients who had fallen (“fallers”) and those who  had not (“nonfallers”). Statistical analysis | Total patients (N=7921). of Which fallers were (n=96). They were male (n=52, 54.2%). | Patients over 60 years (n=30, 31.3%). | Patients who fell were: Schizophrenia (n=52), Manic episode (n=7), depressive episode (n=22), organic mental disorder (n=12), alcohol dependence (n=2), mental retardation (n=1). |
| Lavsa et al. 2010 | To investigate the association between  medications and other variables with  increased fall risk among psychiatric  inpatients. | USA | Retrospective case-control study | Chart review of incident reports and medical charts. Statistical analysis | Total patients (N=1548) of which fell (n=774). These were women (n=446, 57.6%). | Median age 60, 18-39 (n=148, 19.1%), 40-64 (n=299, 38.6%), 65+ (n=327, 42.2%). | Major depressive disorder (n=294, 38%), Schizophrenia/schizoaffective (n=178, 23%), Bipolar (n=161, 20.8%), OCD (n=122, 15.7%), Dementia or Alzheimer (n=102, 13.8%), Anxiety or neurosis (n=88, 11.4%), personality disorder (n=74, 9.6%). |
| Estrin et al. 2009 | To  add to the existing research on fall  risk among psychiatric inpatients in  an acute setting. | USA | Retrospective case-control study | Retrospective medical record review of patients who fell. | Total patients (N=148), of which fallers (n=74), of them most were female (n=47). | Of patients who fell the age was (M 41, SD 17.3) for male and (M 41.9, SD 19.1) for female. | Diagnosis were included for all patients (N=148) were bipolar (n=36, 24%), schizophrenia or psychotic disorder (n=26, 18%), major depressive disorder (n=26, 18%) and opiate dependence (n=10, 7%). |
| de Carle & Kohn. 2001 | To identify risk factors associated with falls in a psychogeriatric inpatient population. | USA | Retrospective cohort study | Analysis of computerized hospital records. Statistical analysis. Mutiple regression analyses. | Total sample of patients (N=1834), of (n=175) fell. Patients who fell were female (79.4%). | 60-69 age group was 24.6%, 70-79 (40%) 80+ (35.4%). | Diagnosis of patients who fell Affective disorder (59.4%) Dementia disorder (50.3%), psychotic disorder (8%). |
| Tseng et al. 2013 | To  identify predictors of falls, and to measure the  severity of injuries among psychiatry inpatients | Taiwan | Retrospective case-control study | Retrospective analysis of incident reports. Statistical analysis | Total sample (N=5894) of which fell (n=545, 9.2%) of which (n=397, 72.8%) were male. | The average age was 42.2 (SD 14.5), with age groups 20-29 (n=135, 24.8%), 30-39 (n=94, 17.2%), 40-49 (n=134, 24.6%), 50-59 (n=125, 22.9%), 60+ (n=57, 10.5%). | Diagnosis were Schizophrenia (n=327, 60%), bipolar disorder (n=97, 17.8%), Dementia (n=21, 3.9%), Substance dependence (n=8, 1.5%), neurosis (n=92, 16.9%). |

**Table 2.** Quantitative synthesis

| Study | Incidence of fall | Risk factor for fall | Mechanism of fall |
| --- | --- | --- | --- |
| Vaughn et al. 1993 | 3.7 falls per 1.000 patient days | Female gender  Diagnosis of depression  Being anxious  Being agitated  Orthostatic hypotension  Dizziness  Syncope | Not reported |
| Yates & Tart 2012 | 7.97 falls per 1.000 patient days | Being alert  Being confused | Not reported |
| Aso & Okamura 2019 | 13.3% (N=120) | History of falls  Increased Body sway (Romberg test) | Not reported |
| Scanlan et al. 2012 | 1.25 per 1000 patient days | Wet floor  Equipment  Trip/stumble  Patient behavior  Footwear / socks  Rushing  Not using mobility aide  Balance / mobility difficulties  Dizziness  Effects of medication  Medical condition  Confusion / mental state  Substance intoxication / withdrawal  Toilet urgency | Fall while walking  Standing  Sitting  Attempting to stand  Getting out of bed |
| Suga et al. 2019 | 1.57 per 1000 patient days | Gait / ambulation  Patients over 65  Wheelchair / walker  Unsteady gait  Requiring help for transfer  Patients less than 65  Side effects of medication  Unsteady gait  Short-stepped gait  Forward head posture | Not reported |
| Struble-Fitzsimmons 2018 | Not reported | Increased Time Up and Go (TUG) time | Not reported |
| Tsuji et al. 2017 | Not reported | Shorter one leg standing time  Time Up and Go (TUG) time  Sialorrhea (DIEPSS) | Not reported |
| Meng et al. 2010 | Not reported | Elderly patient  mood disorder  co-morbidity (medical conditions).  Young females with eating disorder | Slip or trip (n=11, 41%)  Dizziness (n=6, 22%)  Losing balance (n=5, 19%)  Muscle weakness (n=2, 7%)  Used bad support (n=1, 4%)  Knocked against wall (n=1, 4%) |
| Blair & Gruman 2006 | Not reported | High dose antipsychotic  Ambulating | Ambulating  Getting to/from chair  Getting to/from bed  Getting to bathroom  Showering  Exercising |
| Healey et al. 2007 | 2.1 per 1000 patient days | Being old (85-89 years) | Not reported |
| Oepen et al. 2018 | 17.7 per 1000 patient days | Mood disorder  Cognitive impairment (dementia) | Not reported |
| Tsai et al. 1998 | 6.25% (N=192) | Confusion / Disorientation  Elimination problems  History of falls  Difficulty with mobility in lower extremities  Temperature elevation  Generalized weakness | Getting out of bed  Sitting to standing  Walking to bathroom |
| Nyberg et al. 1997 | 17.1 per 1000 patient days | Being female  Being over 80 years of age | Not reported |
| Furness et al. 2017 | 14.5% (N=482) | Depression  Dementia  Psychotic illness  Being older (78.2 vs 74.9)  Postural hypotension  Blood glucose > 5.5mmol/L  Narrow base of support  Curvature of thoracic spine  History of fall  Patient reported intrinsic factors  Feeling dizzy  Losing balance | Not reported |
| Jones et al. 1991 | Not reported | Affective disorder  Substance-dependence  Neurotic personality disorder  Schizophrenic disorder  Childhood disturbances | Not reported |
| Abraham 2012 | Not reported | Intrinsic factors  Patient gait  History of falls  multiple medications  Extrinsic factors  Teamwork  Physical therapy evaluation  Supervision  Toileting | Not reported |
| Irvin 1999 | Not reported | Gait/balance  History of falls | Not reported |
| Draper et al. 2004 | 29% (N=95) | Fall in the previous week  Behavioural overactivity  Agitation  Electroconvulsive therapy (ECT) | Not reported |
| Chan et al. 2013 | 1.1 per 1000 patient days | Being older  Schizophrenia spectrum disorder  Bipolar disorder  Manic episode  Psychiatric comorbidities  History of medical problems  Fall in the past 6 months  Agitation  Disorientation  Impaired consciousness  Unstable gait  Dizziness  Abnormal blood pressure  Restricted mobility  Lower limb problems  More severe psychiatric symptoms  More severe subjective EPS symptoms  Parkinsonism scores (ESRS)  Higher dose of alprazolam  Benzodiazepine  Mood stablizers  Anti-hypertension agents | Ambulating  Changing position  Using toilet  Getting out of bed  Sleeping or reposition |
| Knight et al. 2010 | Not reported | Psychotic disorder  Complex medication regimen  Upward titaration of antipsychotic drugs  Side-effect of medication (dizziness, sedation, light-headedness)  Drop in blood preassure (hypotension)  Elevated postural pulse | Not reported |
| Dickinson et al. 2014 | 2.9 per 1000 patient days | Not reported | While mobilizing  Slip, trip  Fall from bed  Fall from chair  Faint / dizziness / collapsed  Fall from toilet  Fall from wheelchair  Assisted to floor  Unassisted transfer  Fall from commode  Assisted transfer  While being transported |
| Brown & Edelstein 2000 | Not reported | Psychotic disorder  Antipsychotic medication  Cardiovascular disorder | Not reported |
| Feng-Rong et al. 2009 | 1.2% of all inpatients* | Male gender  Age above 60  Length of illness > 10 years  Organic mental disorder  Mental retardation  Depressive episode  Schizophrenia  Clozapine  SSRIs  Mood stabilizers  Benzodiazepines  Psychotropic polypharmacy | Standing up  Getting out of bed  Walking  Walking to bathroom  Extended standing |
| Lavsa et al. 2010 | Not reported | Bipolar disorder  dementia  Alzheimer  Psychotropic agents  Atypical antipsychotics  Conventional antipsychotics  Benzodiazepines  SSRIs  Atypical antidepressants  Anticonvulsants or mood stabilizers  Lithium  Cardiovascular agents  ACE inhibitors  Beta-blockers  Miscellaneous agents  Oral hypoglycemics  Laxatives or stool softeners  Histamine H2 blockers | Not reported |
| Estrin et al. 2009 | Not reported | Bipolar disorder  Age over 60  Physical symptoms on the day of the fall  More medical history items  More medication  Urinary frequency  Incontinence  Generalized weakness  History of falls (past 90 d)  History of syncope  History of impaired mobility  Antihypertensive medication  Benzodiazepine (clonazepam)  Mental status impairment  Dizziness  Unsteady gait | Not reported |
| de Carle & Kohn 2001 | 9.5% fall rate | Female gender  Receiving ECT  Dementia  More medical problems  Dopamine agonist  Antipsychotics  Length of stay (days)  Parkinson’s disease | Not reported |
| Tseng et al. 2013 | 9.2% fall rate | Age over 60 years  Male gender  Bipolar disorder  Schizophrenia  Dementia  Substance abuse | Not reported |

**Table 3.** Qualitative synthesis

|  | Study ID | | |
| --- | --- | --- | --- |
|  | Ocker et al. 2020 | Bayramzadeh et al. 2019 | Lee et al. 2012 |
| Incidence of fall | n/a | Geropsychiatric 6.9 per 1000 patient days | n/a |
|  |  | Alcohol substance unit 6.8 per 100 patient days |  |
|  |  | Adult psychiatric unit 4.9 per 1000 patient days |  |
| Risk for fall | Environmental factors  Wet floor  Tripping on blanket  Cold room temperature  Lack of patient visibility from nurses’ station  Fall Risk Assessment  Inconsistency in RN fall risk assessment  High-risk fall patients not identified by RN’s  Patient factors  Incontinence  Confusion memory deficits  Gait, balance limitations  Medications  Staff communication  Rapid response team not called postfall  , sLack of team communication regarding patient fall risk and interventions | Extrinsic (physical environment)  -Workspace envelope  (Workspace envelope is defined  as physical attributes of the environment)  -Ambient environment  -Environment (products)  Intrinsic  Disease  History of falls  Mobility deficiencies  s deficiencies  other psychological factors  physiological factors | Rules, policies and procedures  Current system for fall assessment needs improvement  Management of medication needs improvement  Lack of specific intervention for a particular patient or patient population  Current system for falls intervention needs improvement  Environment/equipment  Environment needs improvement  Lack/inappropriate use of equipment  Treatment area or milieu problems  Communication  Verbal communication of fall risk needs improvement  Documentation needs improvement  Medical record needs improvement  Patient characteristics  Medical issues  Patient culture  Need for staff education  Fatigue/scheduling problems  Facility characteristics |
| Mechanism of fall | n/a | n/a | Getting up  Walking or running  Bathroom related  Behaviour related |
