# Supplementary material for: Patient Falls in Seclusion Rooms in Psychiatric Inpatient Care: A Sociotechnical Probabilistic Risk Modeling Study
Source: J Nurs Care Qual. 2022 Dec 7;38(2):190–7. doi: 10.1097/NCQ.0000000000000683 (PMC9944373; doi:10.1097/NCQ.0000000000000683)
Supplement: SUPPLEMENTARY MATERIAL [file jncqu-38-190-s002.docx]

**Supplemental Digital Content Table.** Minimal cut sets with highest probability using average estimates

| Cut set | Contributed probability | Risk factors and probability estimates | | | |  |  |
| --- | --- | --- | --- | --- | --- | --- | --- |
| 1. | 0.0000835266 | Schizophrenia (0.1244) | Risk assessment failed (0.7) | Intervention not conducted or failed (0.1) | Getting out of bed (0.056390) | Narrow base of support (0.17010) |  |
| 2. | 0.00008083076 | Schizophrenia (0.1244) | Risk assessment failed (0.7) | Intervention not conducted or failed (0.1) | From seated to standing position (0.054570) | Narrow base of support (0.17010) |  |
| 3. | 0.00007071035 | Schizophrenia (0.1244) | Risk assessment failed (0.7) | Intervention not conducted or failed (0.1) | Getting out of bed (0.056390) | Obesity (0.1440) |  |
| 4. | 0.00006928633 | Schizophrenia (0.1244) | Risk assessment failed (0.7) | Intervention not conducted or failed (0.1) | Getting out of bed (0.056390) | Benzodiazepines (0.14110) |  |
| 5. | 0.00006842816 | Schizophrenia (0.1244) | Risk assessment failed (0.7) | Intervention not conducted or failed (0.1) | From seated to standing position (0.054570) | Obesity (0.1440) |  |
| 6. | 0.00006705009 | Schizophrenia (0.1244) | Risk assessment failed (0.7) | Intervention not conducted or failed (0.1) | From seated to standing position (0.054570) | Benzodiazepines (0.14110) |  |
| 7. | 0.00006594723 | Schizophrenia (0.1244) | Risk assessment failed (0.7) | Intervention not conducted or failed (0.1) | Getting out of bed (0.056390) | Stool softeners (0.13430) |  |
| 8. | 0.00006381876 | Schizophrenia (0.1244) | Risk assessment failed (0.7) | Intervention not conducted or failed (0.1) | From seated to standing position (0.054570) | Stool softeners (0.13430) |  |
| 9. | 0.00005995921 | Bipolar disorder (0.0893) | Risk assessment failed (0.7) | Intervention not conducted or failed (0.1) | Getting out of bed (0.056390) | Narrow base of support (0.17010) |  |
| 10. | 0.00005892529 | Schizophrenia (0.1244) | Risk assessment failed (0.7) | Intervention not conducted or failed (0.1) | Getting out of bed (0.056390) | Gait abnormality (0.12000) |  |
